# Silver Nanoparticles as Potential Antiviral Agents

**DOI:** 10.3390/molecules16108894

**Published:** 2011-10-24

**Authors:** Stefania Galdiero, Annarita Falanga, Mariateresa Vitiello, Marco Cantisani, Veronica Marra, Massimiliano Galdiero

**Affiliations:** 1 Department of Experimental Medicine, II University of Naples, Via De Crecchio 7, 80138, Naples, Italy; E-Mails: mteresa.vitiello@unina2.it (M.V.); veronica_marra@hotmail.it (V.M.); 2 Department of Biological Sciences, Division of Biostructures, Via Mezzocannone 16, 80134, Naples, Italy; E-Mails: sgaldier@unina.it (S.G.); annarita.falanga@unina.it (A.F.); marco.cantisani@unina.it (M.C.); 3 CIRPeB, Department of Biological Sciences, - Via Mezzocannone 16, 80134, Naples, Italy; 4 IBB CNR, CNR, Via Mezzocannone 16, 80134, Naples, Italy

**Keywords:** silver nanoparticles, virus infection, antiviral therapy

## Abstract

Virus infections pose significant global health challenges, especially in view of the fact that the emergence of resistant viral strains and the adverse side effects associated with prolonged use continue to slow down the application of effective antiviral therapies. This makes imperative the need for the development of safe and potent alternatives to conventional antiviral drugs. In the present scenario, nanoscale materials have emerged as novel antiviral agents for the possibilities offered by their unique chemical and physical properties. Silver nanoparticles have mainly been studied for their antimicrobial potential against bacteria, but have also proven to be active against several types of viruses including human imunodeficiency virus, hepatitis B virus, herpes simplex virus, respiratory syncytial virus, and monkey pox virus. The use of metal nanoparticles provides an interesting opportunity for novel antiviral therapies. Since metals may attack a broad range of targets in the virus there is a lower possibility to develop resistance as compared to conventional antivirals. The present review focuses on the development of methods for the production of silver nanoparticles and on their use as antiviral therapeutics against pathogenic viruses.

## 1. Introduction

Viruses represent one of the leading causes of disease and death worldwide. Thanks to vaccination programmes, some of the numerous diseases that used to kill many and permanently disable others have been eradicated, such as smallpox in 1979 [1] or have greatly reduced the burden of the disease, such as in the case of the paralytic disease poliomyelitis [2]. However, for some of today’s most pressing viral pathogens, there is still no vaccine available. To realize the huge economic impact that several viral diseases cause to the global community, we need only to think to common colds, influenza, various problems due to herpesviruses (from shingles, genital herpes, chickenpox, infectious mononucleosis, up to herpes keratitis, neonatal disseminated infections, or viral encephalitis). Other viruses are also able to cause considerable distress and sometimes persistent infections that may lead to cancer or to acquired immunodeficiencies, such as hepatitis viruses (mainly HBV and HCV) or human immunodeficiency virus (HIV). Much effort has been expended in attempts to develop vaccines for these diseases, without appreciable success, at least for some of these viruses, namely, HCV, HIV and some herpesviruses. Presently, the development of new vaccines for such viruses seems likely to continue to be elusive. Together with the risk of emerging or re-emerging viral agents, the field of antiviral compound discovery is very promising.

Emerging and re-emerging viruses are to be considered a continuing threat to human health because of their amazing ability to adapt to their current host, to switch to a new host and to evolve strategies to escape antiviral measures [3].

Viruses can emerge because of changes in the host, the environment, or the vector, and new pathogenic viruses can arise in humans from existing human viruses or from animal viruses. Several viral diseases that emerged in the last few decades have now become entrenched in human populations worldwide. The best known examples are: SARS coronavirus, West Nile virus, monkey pox virus, Hantavirus, Nipah virus, Hendravirus, Chikungunya virus, and last but not least, the threat of pandemic influenza viruses, most recently of avian or swine origin. Unfortunately the methodological advances that led to their detection have not been matched by equal advances in the ability to prevent or control these diseases. There have been improvements in antiviral therapy, but with a wide margin of ineffectiveness, therefore new antiviral agents are urgently needed to continue the battle between invading viruses and host responses. Technological advances have led to the discovery and characterization of molecules required for viral replication and to the development of antiviral agents to inhibit them. Most viruses are, indeed, provided by an extraordinary genetic adaptability, which has enabled them to escape antiviral inhibition and in certain cases to regain advantage over the host by mutagenesis that create new viral strains with acquired resistance to most of the antiviral compounds available [3].

**Figure 1:**
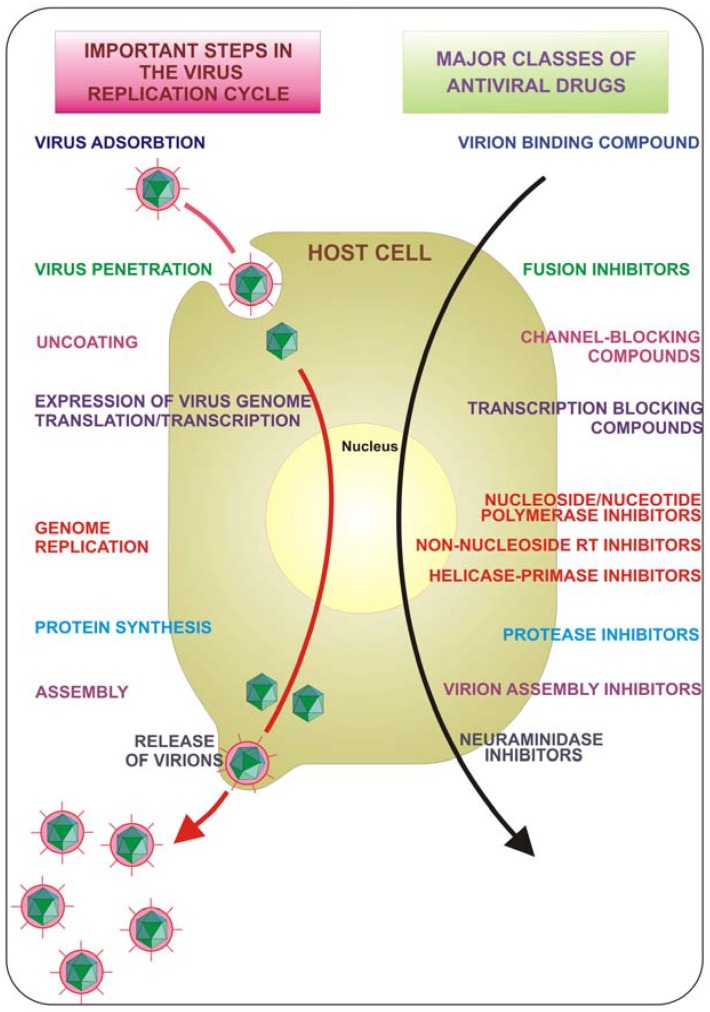
Key steps in the virus replication cycle that provide antiviral targets.

The course of viral infections is governed by complex interactions between the virus and the host cellular system. All viruses depend upon a host cell for their protein synthesis. Thus, all viruses replicate via a broadly similar sequence of events (Figure 1). The virus must first bind to the cell, and then the virus or its genome enters in the cytoplasm. The genome is liberated from the protective capsid and, either in the nucleus or in the cytoplasm, it is transcribed and viral mRNA directs protein synthesis, in a generally well regulated fashion. Finally, the virus undergoes genome replication and together with viral structural proteins assembles new virions which are then released from the cell. Each of the single described phases represents a possible target for inhibition. Drugs that target viral attachment or entrance have proved to be very difficult to be discovered. In fact, to date, only one entry inhibitor has been approved by the US Food and Drug Administration (FDA). This is enfuvirtide (T-20), a synthetic peptide that targets the HIV gp41 envelope protein to prevent fusion. 

Targeting the early steps of virus entry is a very attractive strategy for therapeutic intervention since the site of action of the inhibitor is likely to be extracellular and therefore relatively accessible; this could be paired by a concomitant action of the same drug on multiple targets to obtain a more effective therapeutic compound. Moreover one could expect, in the future, antiviral agents with a broad-spectrum of action against viruses of different families, to be used as first aid compounds against unforeseen viral epidemics or pandemics.

Due to the outbreak of the emerging infectious diseases caused by different pathogenic viruses and the development of antiviral resistance to classical antiviral drugs, pharmaceutical companies and numerous researchers are seeking new antiviral agents. In the present scenario, nanoscale materials have emerged as novel “antimicrobial agents” due to their high surface area to volume ratio and their unique chemical and physical properties [4,5].

Nanotechnology is an emerging field of applied science and cutting edge technology that utilizes the physico-chemical properties of nanomaterials as a means to control their size, surface area, and shape in order to generate different nanoscale-sized materials. Among such materials, metal-based ones seem the most interesting and promising, and represent the subject of the present review. Nanotechnology is directly linked with physics, chemistry, biology, material science and medicine. In fact, it finds application in multiple aspects of research and in everyday life such as electronics and new material design. However, its use in medical research is probably one of the fastest growing areas in which the functional mechanisms of nanoparticles and especially metal-based nanoparticles are just beginning to be exploited. Nanotechnologies have been used to develop nanoparticle-based targeted drug carriers [6,7], rapid pathogen detection [8,9], and biomolecular sensing [10], as well as nanoparticle-based cancer therapies [11,12]. The use of nanoparticles can be extended to the development of antivirals that act by interfering with viral infection, particularly during attachment and entry.

Nanoparticles are properly defined as particles with at least one dimension less than 100 nm, and have attracted much attention because of their unique and interesting properties. Their singular physical (e.g., plasmonic resonance, fluorescent enhancement) and chemical (e.g., catalytic activity enhancement) properties derive from the high quantity of surface atoms and the high area/volume relation, in fact, as their diameter decreases, the available surface area of the particle itself increases dramatically and as a consequence there is an increase over the original properties of their bulk materials.

Considering that biological interactions are generally multivalent, the interplay between microbes and host cells often involves multiple copies of receptors and ligands that bind in a coordinated manner, resulting in enhanced specificities, efficiencies, and strengths of such interactions that allow the microbial agent to take possess of the cell under attack. The attachment and entry of viruses into host cells represent a terrific example of such multivalent interactions between viral surface components and cell membrane receptors [13]. Interfering with these recognition events, and thereby blocking viral entry into the cells, is one of the most promising strategies being pursued in the development of new antiviral drugs and preventive topical microbicides [14,15,16]. In recent years, the use of metal nanoparticles, that may or not have been functionalized on their surface for optimising interactions, is seeing increasing success. The idea of exploiting metals against microorganisms can be considered ancient; in fact, the use of silver was a common expedient for cooking procedures and for preserving water from contamination. The importance of silver for its curative properties has been known for centuries, in fact, silver has been the most extensively studied metal for purpose of fighting infections and preventing food spoilage, and notwithstanding the decline of its use as a consequence of the development of antibiotics, prophylaxis against gonococcal ophthalmia neonatorum with silver ions was considered the standard of care in many countries until the end of the 20th century [17]. Silver’s mode of action is presumed to be dependent on Ag^+^ ions, which strongly inhibit bacterial growth through suppression of respiratory enzymes and electron transport components and through interference with DNA functions [18]. Therefore, the antibacterial, antifungal and antiviral properties of silver ions and silver compounds have been extensively studied. Silver has also been found to be non-toxic to humans at very small concentrations. The microorganisms are unlikely to develop resistance against silver as compared to antibiotics as silver attacks a broad range of targets in the microbes. Considering the broad literature that describes silver, as a bulk material, effective against a wide range of pathogens, silver nanoparticles have been analysed and found to be extremely appealing. The silver nanoparticles have also found diverse applications in the form of wound dressings, coatings for medical devices, silver nanoparticles impregnated textile fabrics [19]. The advantage of using silver nanoparticles for impregnation is that there is continuous release of silver ions enhancing its antimicrobial efficacy. The burn wounds treated with silver nanoparticles show better cosmetic appearance and scarless healing [20]. Silver nanoparticles have received considerable attention as antimicrobial agents and have been shown to be effective mainly as antibacterial. Antimicrobial effectiveness was shown for both Gram-positive and Gram-negative bacteria [21,22].

The antibacterial activity of silver nanoparticles was mainly demonstrated by *in vitro* experiments. Activity against methicillin-resistant *Staphylococcus aureus* (MRSA) [23], *Escherichia coli* [4,21,24,25], *Pseudomonas aeruginosa* [4], *Vibrio cholera* [4] and *Bacillus subtilis* [25] has been documented. Low concentrations of silver nanoparticles were able to consistently inhibit *E. coli* [5] while the growth-inhibitory effect on *S. aureus* was minor. Synergistic antimicrobial activity of silver or zinc nanoparticles with ampicillin, penicillin G, amoxicillin, kanamycin, erythromycin, clindamycin, chloramphenicol and vancomycin against *S. aureus*, *E. coli, Salmonella typhi* and *Micrococcus luteus* was observed [26,27,28].

Also gold nanoparticles have been exploited as antimicrobial agents, mainly as a tool to deliver other antimicrobials or in order to enhance the photodynamic killing of bacteria [29]. Many studies have shown the antimicrobial effects of metal nanoparticles, but the effects of silver nanoparticles against fungal pathogens are mostly unknown; silver nanoparticles, indeed, showed significant antifungal activity against *Penicillium citrinum* [30], *Aspergillus niger* [30], *Trichophyton Mentagrophytes* [31] and *Candida albicans* [32].

Different types of nanomaterials like copper, zinc, titanium [33], magnesium, gold [34], alginate [35] and silver have come up in recent years and most of them have proven to be effective against diverse microorganisms.

The present review aims at a description of the reported antiviral activities of metal nanoparticles and their production methods, with particular regard to silver nanoparticles.

## 2. Metal Nanoparticles and Antiviral Activity

Metal nanoparticles have been studied for their antimicrobial potential and have proven to be antibacterial agents against both Gram-negative and Gram-positive bacteria [4,5,21,26,36]. Theoretically, any metal could be analysed for antiviral activity, however, little effort has been done to determine the interactions of metal nanoparticles with viruses, and only recently some studies have emerged showing that metal nanoparticles can be effective antiviral agents against HIV-1 [37,38,39,40], hepatitis B virus [41], respiratory syncytial virus [42], herpes simplex virus type 1 [43,44], monkeypox virus [45], influenza virus [46] and Tacaribe virus [47].

Seen the paucity of viruses that have been investigated and the fact that most of the nanoparticles used were made of silver, this section will be instrumental to analyse the inhibitory effect for each single virus (Table 1).

### 2.1. Retroviridae

Acquired immunodeficiency syndrome (AIDS), the disease caused by HIV, is responsible for over two million deaths per year, among more than 33 million people that are infected. Highly active anti-retroviral therapy (HAART), a treatment regimen that employs a cocktail of drugs to suppress HIV infection, has significantly improved the quality of life and life expectancy of millions of HIV-infected individuals. Numerous HIV-infected individuals are currently treated with HAART, and these individuals harbor chronic long-term infection; as a result, HIV eventually develops resistance to these drugs, resulting in a need to change medication regimens and a subsequent increase in the cost of treatment [48].

The replication cycle of HIV-1 is a complex multistep process that depends on both viral and host cell factors. Entry into target cells is achieved through fusion of the viral lipid envelope and the cellular plasma membrane [49]. The viral component that acts as a fulcrum for mediating fusion is the trimeric envelope glycoprotein composed of two subunits: gp120, which binds to the cellular receptor, and gp41, which is the subunit bearing the transmembrane segment, and that executes fusion [50]. Following gp120 binding to the cellular receptor, CD4, and a subsequent interaction with CCR5 or CXCR4 co-receptors, a conformational change of gp41 leads to membrane fusion and delivery of the capsid to the cytoplasm. Soon after entry, the RNA is reverse-transcribed into a complementary DNA which is converted to a double-stranded DNA, and integrated into the cellular genome. The integrated proviral DNA is transcribed to generate full-length progeny viral RNA and a number of spliced mRNA transcripts. Transcription and translation, performed by the cellular machinery, result in the synthesis of viral proteins that together with the progeny viral RNA are transported to the site of virus particle assembly at the plasma membrane, where the virus gains access to the extracellular milieu upon budding events [51].

Elechiguerra *et al*. [37] were the first to describe the antiviral activity of metal nanoparticles, in fact, they found that silver nanoparticles undergo size-dependent interactions with HIV-1. In their investigations, they explored the possibility that physicochemical properties of nanoparticles may depend on the nanoparticle interactions with a capping agent molecule. For this reason they tested silver nanoparticles with three different surface chemistries: foamy carbon, poly(N-vinyl-2-pyrrolidone) (PVP), and bovine serum albumin (BSA). Foamy carbon-coated silver nanoparticles were embedded in a foamy carbon matrix needed to preclude coalescence during their synthesis. PVP-coated nanoparticles were synthesized using glycerine as both reducing agent and solvent. In this method, a metal precursor is dissolved in a liquid polyol in the presence of a capping agent such as PVP [52]. Synthesis in aqueous solution was performed for BSA-conjugate silver nanoparticles. Interactions of silver nanoparticles with HIV-1 were probed with the aid of high angle annular dark field (HAADF) scanning transmission electron microscopy technology. It was possible to obtain sufficient data to determine that the interaction between HIV particles and silver nanoparticles is clearly due to the size of the silver nanoparticle since only nanoparticles within the range of 1–10 nm were able to bind to the virus. In particular, nanoparticles were not randomly attached to the virus, but all the three species of nanoparticles established regular spatial relationships with the viral envelope. The most probable sites for interaction were found to be the sulfur-bearing residues of the gp120 glycoprotein knobs, which being limited in number, may also explain the inability of larger nanoparticles to bind the virus. The capacity of silver nanoparticles to inhibit infectivity of a laboratory-adapted HIV-1 strain at non-cytotoxic concentrations was determined by *in vitro* assays, and a dose-dependant inhibition of viral infectivity was reported. In particular, BSA- and PVP-coated nanoparticles showed to possess a slightly lower inhibition efficacy, probably because the surface of the nanoparticle is directly bound to and encapsulated by the capping agent. In contrast, the silver nanoparticles released from the carbon matrix have a greater inhibitory effect due to their essentially free surface area. These findings, however, only provide indirect evidence for their proposed mode of interaction through the binding to gp120, therefore, a panel of different *in vitro* assays was used to elucidate the silver nanoparticles mode of antiviral action against HIV-1 [39]. A luciferase-based assay showed that silver nanoparticles coated with PVP were an effective virucidal agent against cell-free virus (including laboratory strains, clinical isolates, T and M tropic strains, and resistant strains) and cell-associated virus. The concentration of silver nanoparticles at which infectivity was inhibited by 50% (IC50) ranged from 0.44 to 0.91 mg/mL. The observed antiviral effect of silver nanoparticles was due to the nanoparticles, rather than just to the silver ions present in the solution. In fact silver salts exerting antibacterial effect through silver ions, inhibited HIV-1 with a therapeutic index 12 times lower than the one of silver nanoparticles. Silver nanoparticles inhibit the initial stages of the HIV-1 infection cycle by blocking adsorption and infectivity in a cell-fusion assay. The inhibitory activity of silver nanoparticles against the gp120-CD4 interaction was also investigated in a competitive gp120-capture ELISA, which together with the cell-based fusion assay, showed that silver nanoparticles inhibit HIV-1 infection by blocking viral entry, particularly the gp120-CD4 interaction. Besides, silver nanoparticles inhibit post-entry stages of the HIV-1 life cycle, in fact, the antiviral activity was maintained also when the metal nanoparticles were added 12 h after the cell had been infected with HIV. Since silver ions can form complexes with electron donor groups containing sulfur, oxygen, or nitrogen that are normally present as thiols or phosphates on amino acids and nucleic acids they might inhibit post-entry stages of infection by blocking HIV-1 proteins other than gp120, or reducing reverse transcription or proviral transcription rates by directly binding to the RNA or DNA molecules. Silver nanoparticles proved to be virucidal to cell-free and cell-associated HIV-1 as judged by viral infectivity assays. HIV infectivity was effectively eliminated following short exposure of isolated virus to silver nanoparticles. These properties make silver nanoparticles a potential broad-spectrum agent not prone to inducing resistance that could be used preventively against a wide variety of circulating HIV-1 strains. PVP-coated silver nanoparticles, being an interesting virucidal candidate drug, have been further investigated as a potential topical vaginal microbicide to prevent transmission of HIV-1 infection [40] using an *in vitro* human cervical tissue-based organ culture that simulates *in vivo* conditions [53,54]. When formulated into a non-spermicidal gel (Replens) at a concentration of 0.15 mg/mL, PVP-coated silver nanoparticles were not toxic to the explant, even when the cervical tissues were exposed continuously to the metal for 48 hours, but one minute of pre-treatment of the cervical explant with 0.025 to 0.15 mg/mL of PVP-coated silver nanoparticles prevented the transmission of cell-associated HIV-1 and cell-free HIV-1 isolates. When pre-treatment was carried on for 20 minutes followed by extensive washing the drug conferred almost total protection against HIV-1 transmission for 48 hours, indicating a long-lasting protective effect by the PVP-coated silver nanoparticles in the cervical explants.

A different group [38] also reported about the antiviral activity of silver nanoparticles that had been fabricated using Hepes buffer. They showed that silver nanoparticles exhibited potent cytoprotective and post-infected anti-HIV-1 activities (at 50 mM a 98% reduction was achieved) toward Hut/CCR5 cells in a dose-dependent fashion. Similar inhibitory activities were reported for the silver nanoparticles when a citrate solution with NaBH_4_ as the reducing agent was used, while lower activity was observed for gold nanoparticles (10 nm, fabricated in Hepes buffer).

### 2.2. Herpesviridae

The herpesvirus family consists of more than 100 double-stranded DNA viruses divided into α, β and γ subgroups. Only eight herpesviruses are known to commonly infect humans and the remainder are animal herpesviruses infecting a wide variety of animal species. All members of the herpesvirus family cause life-long latent infections and, structurally, all have a linear, double-stranded DNA genome packaged into an icosahedral capsid and covered by a lipid envelope with embedded proteins and glycoproteins [55]. Symptomatic diseases caused by HSV-1 (prototypic α-herpesvirus) are generally limited to cold sores of the mouth and keratitis in the eyes, but HSV-1 is capable of causing life-threatening diseases in immunocompromised individuals, including newborns, patients with HIV or patients undergoing immunosuppressive treatment. Transmission among humans requires physical contact and after the initial infection, the virus remains latent in neurons, a key feature of α-herpesviruses [56].

HSV entry into host cells marks the first and possibly most critical step in viral pathogenesis [57]. Five viral glycoproteins have been implicated in the viral entry process: gB, gC, gD, gH and gL. All but gC are essential for entry. The initial interaction, or binding to cells, is mediated via interactions of gC and/or gB with heparan sulfate (HS) [58]. The significant reduction of HSV-1 infection in the absence of either viral gC or cell-surface HS [59] point to a key role of gC high-affinity binding to heparan sulfate (HS) on the cell surface. Following binding, HSV entry is achieved through fusion of the lipid bilayer of the viral envelope with a host cell membrane. The core fusion machinery is composed by gB and gH/gL [60,61], in fact, the complete fusion is only achieved when the three proteins act together. Glycoprotein B may act as the premier fusogen [62,63,64], but it seems to need the cooperation of several membranotropic sequences harboured in gH [65,66,67,68,69]. Transcription, replication of viral DNA and assembly of progeny capsids take place within the host nucleus, and then there is a complex mechanism for the exit of the newly assembled viruses from the cell [70].

Baram-Pinto *et al*. have described in two consecutive works [43,44] the potential of exploiting metal nanoparticles for viral inhibition. Their strategy was proved valid against HSV-1, but was probably intended and may prove useful against other viruses, such as papillomaviruses (HPV) and HIV. In fact, their anti-HSV-1 agents are based on the principle that they mimic HS and may compete for the binding of the virus to the cell. Also HIV or HPV use HS as a docking site during infection, therefore the nanoparticles described by Baram-Pinto *et al*. may be useful as a broad topical microbicide for sexually-transmitted viral infections.

Another point of particular interest from their work is the analysis of the significance of the carrier core material, in fact they designed two different metal particles, one made of silver, and the other made with gold, but both with the same coating of mercaptoethane sulfonate (MES) intended to mimic the polysulfonated HS and therefore expected to create a high local concentration of binding molecules for improved inhibitory effect.

The silver- (Ag-MES) and gold- (Au-MES) MES nanoparticles were tested in antiviral assays using the wild-type HSV-1 McIntyre strain. For the inhibition experiments, Vero cells and/or virus solutions were treated with Ag-MES and Au-MES nanoparticles at different time points to analyse the different stages of the viral infection that may be blocked.

Taken together, their results indicate that sulfonate-capped silver and gold nanoparticles inhibit HSV-1 infections by blocking the attachment and thereby the entrance of the virus into the cells and/or by preventing the cell-to-cell spread of the virus.

The inability of soluble MES and unmodified metal nanoparticles to control viral infectivity stressed the importance of spatially oriented functional groups anchored on a nanoparticle core for viral inhibition. At the same time, antiviral activity shown by both Ag-MES and Au-MES nanoparticles suggest the possibility of using alternative carrier core materials as well.

While these results suggest the versatility of the idea of effective viral inhibitions using functionalized nanoparticles, it also indicates that other core materials could also be efficient as long as they are not toxic to the host cells.

### 2.3. Paramyxoviridae

Respiratory Syncytial Virus (RSV) belongs to the family *Paramyxoviridae* and infects the epithelium of the lungs and the respiratory tract causing serious respiratory disease, especially in children and older people. No vaccine or adequate pharmaceutical compounds are available, underlining the need for the development of future RSV treatments.

The RSV genome consists of a single RNA molecule of negative-sense RNA, which encodes, among others, for two surface glycoproteins, which are exposed on the viral envelope. These glycoproteins are the (G) protein, which serve as a receptor binding protein, and the (F) protein, which is responsible for the fusion between the cell membrane and the viral envelope. As within the name itself of the virus, following infection of cells, the F protein is expressed on the surface of cells and fuse adjacent cells, giving rise to syncytia formation, a well characterised cytopathic effect [71].

Sun *et al*. [42] have utilized silver nanoparticles conjugated to various proteins to study the inhibition of RSV infection in HEp-2 cell culture. In their study, the capping agents used for the silver nanoparticles were: (1) poly(N-vinyl-2-pyrrolidone) (PVP); (2) bovine serum albumin (BSA); and (3) a recombinant F protein from RSV (RF 412).

The preliminary analysis by Transmission Electron Microscopy yielded interesting results on the interaction between silver nanoparticles with RSV virion particles. BSA-conjugated silver nanoparticles seemed to interact with RSV but without a specific association or spatial arrangement, while RF 412-conjugated silver nanoparticles appeared to be floating freely with no proof of regular attachment. On the other hand, PVP-coated silver nanoparticles were able to bind to the viral surface with a regular spatial arrangement, suggesting a possible interaction with G proteins that are evenly distributed on the envelope of the RSV virion. The hypothesised interpretation for the interaction of PVP-nanoparticles with G proteins is that their small size and uniformity (4–8 nm), compared to the other (BSA and RF 412) coated nanoparticles (3–38 nm) may contribute to the effectiveness of the binding.

Since toxicity is an imperative issue regarding pharmaceutical compounds, the cytotoxicity of each of the nanoparticle conjugates was established using the Trypan Blue Exclusion Assay, and revealed that all of them (BSA-, PVP- and RF 412-silver nanoparticles) showed less than 20% cytotoxicity up to a concentration of 100 μg/mL. Silver nanoparticles have to be regarded as potentially harmful especially when intended for treating a respiratory disease such as RSV infections. Sun *et al*. [42] have, therefore considered that a saturated surface capping composed of a natural biomolecule (BSA) and a biocompatible chemical (PVP) could be able to mask the pure nano-silver surface and thus would reduce toxicity without hampering efficacy. Nevertheless, further studies are needed to validate these *in vitro* data for the use into the clinical setting, and investigation of the toxic effects and fate of nanoparticles after their deposition in the respiratory tract is mandatory for the future development of anti-RSV silver nanoparticles-based therapeutic compounds.

HEp-2 cells were infected with RSV mixed with BSA-, PVP- and RF 412-coated silver nanoparticles and infectivity inhibition was evaluated by microscopic examination for syncytia formation and by immunofluorescence microscopy. Neither BSA- nor RF 412- coated nanoparticles showed any significant inhibition of RSV infection, while PVP-coated silver nanoparticles inhibited RSV infection by 44%. These results led the authors to conclude that when the silver nanoparticles are conjugated to the PVP protein and mixed with RSV, they bind to the G protein on the viral surface and interfere with viral attachment to the HEp-2 cells resulting in the inhibition of viral infection.

### 2.4. Hepadnaviridae

Hepatitis B virus (HBV) is a partially double-stranded DNA virus provided with a lipidic envelope coat. HBV has a strong tropism for hepatocytes, and once it has entered the cell, viral particles migrate to the nucleus where the viral genome is completed to form a covalently closed circular DNA (cccDNA) that serves as the template for the subsequent steps of viral mRNA transcription and formation of the pre-genomic RNA (pgRNA). The pgRNA forms the template for the reserve transcription by the viral-encoded reverse-transcriptase that produce new viral genomes [72]. Nucleotide (adefovir) and nucleoside (lamivudine, entecavir, and telbivudine) analogue inhibitors, which represent approved pharmaceuticals with direct antiviral activity against HBV, target primarily the viral polymerase reverse-transcriptase. Although their effectiveness has been proven, raising drug-resistant HBV strains is fast, therefore limiting the use of these antivirals. Lu *et al*. [41] have analysed monodisperse silver nanoparticles for their ability to inhibit HBV replication. The nanoparticles used in their study were prepared from AgNO_3_ in HEPES and measured dimensions of ~10 nm (Ag10Ns), ~50 nm (Ag50Ns) and ~800 nm (Ag800Ns). Silver nanoparticles with particle diameters of 800 nm were too toxic for being evaluated as antiviral compound, but 10 nm and 50 nm particles showed only a minor toxicity at the concentration able to inhibit HBV replication. In fact, nanoparticles of both sizes showed potent anti-HBV activities. The Ag10Ns reached 38% inhibition at 5 µM and 80% at 50 µM, while the Ag50Ns were slightly more active with 53% and 92% at concentration of respectively 5 µM and 50 µM. In the same paper by Lu *et al*. [41], the activity of silver nanoparticles was also compared to 10 nm gold nanoparticles (Au10Ns) and other silver compounds with silver in different oxidation states, and the overall results showed that the anti-HBV effects of silver nanoparticles are undoubtedly much more pronounced. In conclusion, silver nanoparticles were able to inhibit the production of HBV RNA and extracellular virions probably via a specific interaction between the nanoparticles and the double-stranded DNA of HBV and/or direct binding with viral particles.

### 2.5. Orthomyxoviridae

The influenza virus is a highly contagious pathogen that causes annual epidemics in the human population, and is much feared for its potential to generate new viruses able to jump to humans from different animal species and causing pandemics. Recently, Papp *et al*. [46] have described their studies in which functionalized gold nanoparticles were used to inhibit the influenza virus. This is an orthomyxovirus containing a helical capsid with a genome of eight RNA segments. The capsid is covered by a lipid envelope containing mainly two virally-encoded glycoproteins, namely hemoagglutinin (HA) and neuraminidase (NA), that forms spiky projections on the surface. The virus binds to the cell plasma membrane through an interaction between HA and sialic acid (SA) residues present on glycoproteins and lipids on the surface of the host cell. This is soon followed by a mechanism of receptor-mediated endocytosis that brings the enveloped virus particle inside the cytoplasm but surrounded by a second lipid bilayer besides the envelope, the endosomal one. Inside the late endosome, environment acidification triggers a conformational change of HA, which sets in motion a mechanism of protein (HA) mediated fusion of the endosomal membrane with the viral envelope ending with the release of the nucleoproteins and genome fragments into the cytoplasm [73].

Papp *et al*. [46] strategy was to functionalize gold nanoparticles with sialic acid (SA)-terminated glycerol dendron with the objective to inhibit influenza virus binding to the plasma membrane. Gold nanoparticles of different size were produced: one of 2 nm and a second of 14 nm.

They found that 2 nm had no inhibitory effect on the hemagglutination, used to test the ability of the influenza virus to bind to a target membrane. On the contrary, the 14 nm gold nanoparticles inhibited hemagglutination at concentrations in the nanomolar range, demonstrating that the activity clearly depends on the particle dimension and the spatial distribution of the interacting ligand/receptor molecules. The same trend, with a more pronounced activity was observed in influenza virus inhibition assays where the sialylated particles of 14 nm size were found to be effective for influenza virus inhibition, whereas the 2 nm analogues did not show significant impact. Therefore, they proved that sialic-acid-functionalized gold nanoparticles are able to effectively inhibit viral infection.

### 2.6. Poxviridae

Monkeypox virus (MPV), an orthopoxvirus similar to variola virus, is the causative agent of monkeypox in many species of non-human primates, but it is also a human pathogen with a clinical presentation similar to that of smallpox. MPV is considered a big threat to human life and therefore research is being carried out to develop drugs and therapeutic agents against this virus [74].

Different size nanoparticles were produced by plasma gas synthesis and used by Rogers *et al*. [45] in a plaque reduction assay of MPV. The silver nanoparticles used in this work were 25 (Ag-NP-25), 55 (Ag-NP-55) and 80 (Ag-NP-80) nm, and some nanoparticles were also coated with polysaccharide, 10 (Ag-PS-10), 25 (Ag-PS-25) and 80 (Ag-PS-80) nm nanoparticles. These nanoparticles, at concentrations ranging from 12.5 to 100 μg/mL, were evaluated for MPV inhibitory efficacy using a plaque reduction assay. The main results showed that the Ag-PS-25 (polysaccharide-coated, 25 nm) and Ag-NP-55 (non-coated, 55 nm) exerted a significant dose-dependent inhibition of MPV plaque formation, but the mechanism by which this inhibition occurs has not been further investigated. Poxviruses enter cells by endocytosis or direct fusion at the plasma membrane, and at least 9 or 10 envelope proteins are involved in the entry mechanism, this is followed by a regulated sequence of events that allow virus replication. Many steps in the virus life cycle are still unknown, and this report on the activity of silver nanoparticles is too preliminary to attempt to give a satisfactory explanation of their mechanism of action. Probably the silver nanoparticles may intervene in the early steps of binding and penetration by blocking virus-host cell binding by physical obstruction or, if internalised, they can disrupt intracellular pathways important for virus replication.

Rogers *et al*. [45] also described that AgNO_3_ was active as a MPV inhibitor, but at the concentration of 100 µg/mL its toxic effect on Vero cells impeded the evaluation of the antiviral activity. Interestingly, some of the nanoparticles analysed in the study promoted an increase in the mean number of MPV plaques/well at the highest concentrations used. A potential explanation for these contrasting results could lie in the fact that nanoparticles may tend to aggregate and consequently create on cells areas available for increased contacts between viral particles and the cell membrane, therefore augmenting internalization and plaque formation. However, these data are preliminary and need a more in-depth analysis to draw more significant conclusions.

### 2.7. Arenaviridae

The family Arenaviridae is composed of 18 different species of viruses divided into two antigenic groups, the Old World and New World (Tacaribe complex) groups. The Tacaribe complex, in addition to the Tacaribe virus (TCRV), includes the viral hemorrhagic fever-inducing viruses Junin, Machupo, Guanarito, and Sabia. Considering the high transmissibility by person-to-person via the respiratory route, the lack of diagnostic testing, and therapeutic options limited to ribavirin (not a satisfactory efficacy and easily emergence or resistant strains), the arenaviruses are included in the category A list of potential bio-weapons [75].

TCRV is not a human pathogen, but exhibits a close antigenic relationship with Junin and Guanarito viruses [76], therefore could serve well as a model virus for arenavirus derived diseases without human pathogenic potential and adequate safety for laboratory manipulation.

Speshock *et al*. [47] have recently analysed the activity of two types of silver nanoparticles against TCRV: uncoated (Ag-NP) and polysaccharide coated silver nanoparticles (PS-Ag).

They found that when TCRV was treated with 50 μg/mL, 25 μg/mL and even 10 μg/mL of the 10 nm Ag-NPs significant reduction in the progeny virus titer or no detectable progeny virus was produced. PS-Ag particles showed a similar trend but were not as effective, but toxicity was reduced. Therefore the polysaccharide coating may indeed protect the cell from the toxic effects of the Ag-NPs, but it also appears to interfere with the Ag-NP interaction with TCRV.

Silver nanoparticles seem to interact with TCRV prior to cellular exposure resulting in a decrease in viral infectivity with 10 and 25 nm Ag-NPs, therefore, the authors suggested that the silver nanoparticles may bind to virally-encoded membrane glycoproteins. In fact, TCRV glycoproteins are rich in cysteine residues [77] and silver nanoparticles have been shown to bind easily to the thiol groups [78], which are found in cysteine residues. This interaction can either prevent the internalization of the viral particle by interfering with cellular receptor binding, or may favour the internalization of the silver nanoparticle together with the virus and produce an inhibitory effect on viral replication interfering with the TCRV RNA-dependent RNA polymerase (L protein). Other possible mechanism of action could be related to the fact that the silver nanoparticles bound to the viral glycoproteins may prevent the virus uncoating in the endosome.

Finally, Speshock *et al*. [47] proved that pre-treatment of the cells with silver nanoparticles had no effect on viral replication, therefore they concluded that the Ag-NPs could be inactivating the virus prior to entry into the cell.

### 2.8. Virus Inactivation for Water Treatment

The removal of viruses from water (and the environment in general) is of paramount important for health safety maintenance of our modern society that profoundly relies on water safety for drinking and leisure activities. Pathogenic viruses such as adenovirus, norovirus, rotavirus, and hepatitis A commonly occur in both surface and groundwater sources [79,80,81] and must be effectively inactivated to provide safe water.

Titanium dioxide has attracted much attention as a photocatalyst for water treatment, being resistant to corrosion and non-toxic when ingested. The antibacterial properties of TiO_2_ have been well documented [82,83,84,85] and are attributed to the generation of ROS, especially hydroxyl free radicals and hydrogen peroxide [83,86]. While few studies have investigated the antiviral properties of TiO_2_, its potential for inactivating viruses has been demonstrated [84,87,88]. However, the inactivation rates obtained in most of these studies were extremely low. For example, Cho *et al*. [84] demonstrated only minor removal of bacteriophage MS2 after 2 h of irradiation using P25 TiO_2_ suspended at 1 g/L. The inactivation kinetics needs to be greatly improved in order to provide efficient drinking water disinfection.

Liga *et al*. [89] have hypothesized a possible synergic mechanism occurring between silver and TiO_2_ when silver doped titanium dioxide is used for inactivating microorganisms under UV radiation, therefore they demonstrated that silver doping TiO_2_ greatly enhanced the photocatalytic inactivation of viruses primarily by increasing hydroxyl free radicals production in addition to slightly increasing virus adsorption.

Silver doping significantly enhanced MS2 inactivation by P25 TiO_2_ and the inactivation rate increased with silver content. With silver doped TiO_2_ nanoparticles a considerable removal of MS2 could be obtained in 45 seconds, rendering feasible the goal of achieving virus removal from drinking water using photoreactors exploiting metal nanoparticles.

## 3. Toxicity

Although the continuous evolutions in the field of metal-based nanoparticles for drug delivery, medical imaging, diagnostics, therapeutics and engineering technology, there is a serious lack of information about the impact of metal nanoparticles on human health and environment, probably due to the intrinsic complex nature of nanoparticles that have led to different attitudes on their safety.

Therefore, an important issue in the use of metal nanoparticles is their potential toxicity. For metal nanoparticles to be effective as antiviral pharmaceuticals, it is imperative to gain a better understanding of their biodistribution/accumulation in living systems.

The principal characteristic of metal nanoparticles is their size, which falls in between individual atoms or molecules and the corresponding bulk materials. Particle size and surface area can modify the physicochemical properties of the metal material, but can also influence the reactivity of nanoparticles with themselves or with the cellular environment, leading to different modes of cellular uptake and further processing, leading to adverse biological effects in living cells that would not otherwise be possible with the same material in larger forms. In fact, as particle size decreases, some metal nanoparticles show increased toxicity, even if the same material is relatively inert in its bulk form (e.g., Ag, Au, and Cu).

Apart from size, the biological consequences of metal nanoparticles also depend on chemical composition, surface structure, solubility, shape, and aggregation. All of these parameters can modify cellular uptake, protein binding, translocation to the target site, and most of the biological interactions with the possibility of causing tissue injury. Therefore, in terms of safety, the effect of silver nanoparticles is a major consideration: even if they inhibit viral infections, it would not be beneficial if there are adverse effects to humans or animals. A commonly used strategy to reduce a possible toxicity is to use various capping agents to prevent the direct contact of the metal with the cells.

Potential routes of human exposure to metal nanoparticles used as therapeutic compounds include the gastrointestinal tract, the skin, the lungs, and systemic administration. Considering the use of metal nanoparticles from the point of view of a potential antiviral therapy, it is straightforward that the safest and easiest results can be obtained with topical use of nanoparticles as microbicide for direct viral particles inactivation and/or inhibition of the early steps of the viral life cycle, attachment and entry. Therefore, the dermal route seems the one of major concern. Dermal exposure to metal nanoparticles often takes place when using sunscreen lotions, for example, TiO_2_ and ZnO nanoparticles. In healthy skin, the epidermis provides excellent protection against particle spread to the dermis. However, in presence of damaged skin micrometer-size particles gain access to the dermis and regional lymph nodes. A further concern should be the potential of nanoparticles translocation to the brain via the olfactory nerve as a consequence of the vicinity of the nasal mucosa to the olfactory bulb. Whether nanoparticles in such tissues have any pathological or clinical significance is uncertain, therefore, more data is needed to properly address the safety concern on the use of metal nanoparticles as pharmaceuticals.

Several studies have demonstrated the cytotoxic effects of metal nanoparticles [90,91,92,93], in fact, silver nanoparticles were found to be highly cytotoxic to mammalian cells based on the assessment of mitochondrial function, membrane leakage of lactate dehydrogenase, and abnormal cell morphologies [90,91,92,93]. At a cellular level, metal nanoparticles interact with biological molecules within mammalian cells and can interfere with the antioxidant defence mechanism leading to the generation of reactive oxygen species (ROS). Such species, in excess, can cause damage to biological components through oxidation of lipids, proteins, and DNA. Oxidative stress may have a role in the induction or the enhancement of inflammation through upregulation of redox sensitive transcription factors (e.g., NF-κB), activator protein-1, and kinases involved in inflammation [94,95,96,97].

The generation of reactive oxygen species by cells exposed to silver nanoparticles [91] has been showed in human lung fibroblast and human glioblastoma cells, and as a consequence DNA damage and cell cycle abnormalities have been observed. Accumulation of silver nanoparticles in various organs (lungs, kidneys, brain, liver, and testes) has been evidenced in animal studies [98]. Most of the *in vitro* studies show dose dependence, in fact, higher doses of silver induce a strongher cellular toxicity. Nevertheless, should be considered that *in vitro* concentrations of nanoparticles are often much higher than the ones used in *in vivo* experiments, therefore such exposures do not represent a replica of the conditions expected for *in vivo* exposure. A recent study [99] showed that mice exposed to silver nanoparticles showed minimal pulmonary inflammation or cytotoxicity following subacute exposures, but longer term exposures with higher lung burdens of nanosilver are not investigated, therefore eventual cronic effects may be underscored.

This review presents only a brief description of the toxicity derived from the use of metal nanoparticles. A more detailed coverage of the topic is available in recently published reviews [100,101,102,103]. Although significant progress has been made to elucidate the mechanism of silver nanomaterial toxicity, a proved consensus on the immediate impact or long term effect on human health is still missing. Further research is required to provide the necessary warranties to allow a safely exploitation of the interesting *in vitro* antiviral properties of silver nanoparticles and their transfer to the clinical setting.

## 4. Metal Nanoparticles Production

Nanoparticles are nanoscale clusters of metallic atoms, engineered for some practical purpose, most typically antimicrobial and sterile applications. Different wet chemical methods have been used for the synthesis of metallic nanoparticles dispersions. The early methods to produce nanoparticles of noble-metals are still used today and continue to be the standard by which other synthesis methods are compared.

The most common methods involve the use of an excess of reducing agents such as sodium citrate [104] or NaBH_4_ [105]. Ayyanppan *et al*. [106] obtained Ag, Au, Pd and Cu nanoparticles by reducing metallic salts in dry ethanol. Longenberger *et al*. [107] produced Au, Ag and Pd metal colloids from air-saturated aqueous solutions of poly(ethylene glycol) (PEG). Reduction methods can also be used for the production of Pt, Pd, Cu, Mi *etc*., although specific protocols depend on the reduction potential of the source ion [108]. Cu and Ni are not very stable as the metal particles are easily oxidized requiring strong capping ligands to prevent the oxidation.

Initially, the reduction of various complexes with metallic ions leads to the formation of atoms, which is followed by agglomeration into oligomeric clusters. Controlled synthesis is usually based on a two-step reduction process: in the first step a strong reducing agent is used to produce small particles; in the second step these small particles are enlarged by further reduction with a weaker reducing agent [104]. Strong reductants lead to small monodisperse particles, while the generation of larger size particles can be difficult to control. Weaker reductants produce slower reduction reactions, but the nanoparticles obtained tend to be more polydisperse in size. Different studies reported the enlargement of particles in the secondary step from about 20–45 nm to 120–170 nm [109].

Another general method for the production of different metal nanoparticles (Au, Ag, Pt, Pd) uses commonly available sugars, e.g., glucose, fructose and sucrose as reducing agents [110]. This approach has several important features: (1) sugars (glucose, fructose, and sucrose) are easily available and are used as reducing agents; (2) upon their exploitation no other stabilizing agent or capping agent is required to stabilize the nanoparticles; (3) sugars are very cheap and biofriendly (4) the nanoparticles can be safely preserved in a essiccator for months and redispersed in aqueous solution whenever required instead of being kept in aqueous solution.

An array of other physical and chemical methods have been used to produce nanomaterials. In order to synthesize noble metal nanoparticles of particular shape and size specific methodologies have been formulated, such as ultraviolet irradiation, aerosol technologies, lithography, laser ablation, ultrasonic fields, and photochemical reduction techniques, although they remain expensive and involve the use of hazardous chemicals. Therefore, there is a growing concern to develop environment-friendly and sustainable methods.

Biosynthesis of gold, silver, gold–silver alloy, selenium, tellurium, platinum, palladium, silica, titania, zirconia, quantum dots, magnetite and uraninite nanoparticles by bacteria, actinomycetes, fungi, yeasts and viruses have been reported. However, despite the stability, biological nanoparticles are not monodispersed and the rate of synthesis is slow. To overcome these problems, several factors such as microbial cultivation methods and extraction techniques have to be optimized and factors such as shape, size and nature can be controlled through just modifying pH, temperature and nutrient media composition. Owing to the rich biodiversity of microbes, their potential as biological materials for nanoparticle synthesis is yet to be fully explored. The production of metal nanoparticles involves three main steps, including (1) selection of solvent medium; (2) selection of environmentally benign reducing agent; (3) selection of nontoxic substances for the nanoparticles stability [111].

Biomineralization is also an attractive technique, being the best nature friendly method of nanoparticle synthesis. In one of the biomimetic approaches towards generation of nanocrystals of silver, reduction of silver ions has been carried out using bacteria and unicellular organisms. The reduction is mediated by means of an enzyme and the presence of the enzyme in the organism has been found to be responsible of the synthesis [112,113].

Therefore in search of a methodology that could provide safer and easier synthesis of metal nanoparticles, it seems that the biogenic synthesis using the filtrated supernatant of different bacterial and fungal cultures is having a considerable impact, where the reduction of metal ions occurs through the release of reductase enzymes into the solution [28,114,115,116]. For an extensive coverage of the biological synthesis of metal nanoparticles by microbes, refer to the recent review by Narayanan and Sakthivel [117].

## 5. Conclusions

**Figure 2:**
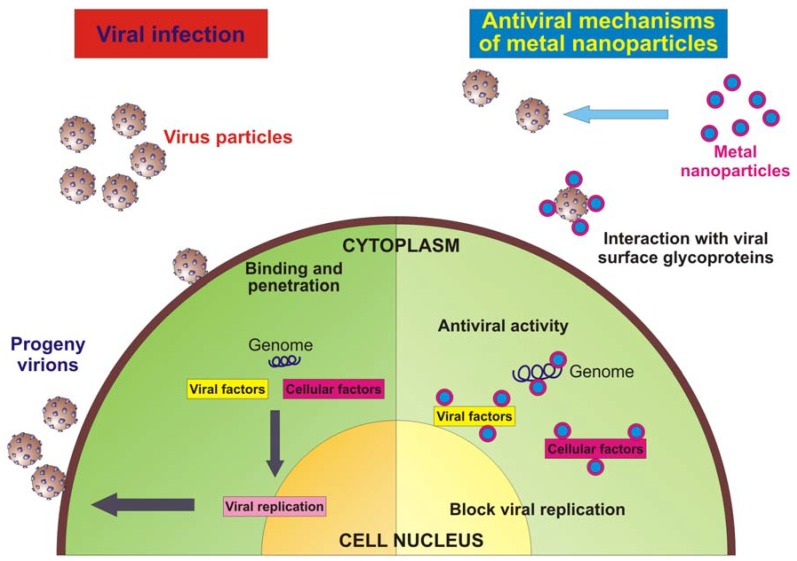
Schematic model of a virus infecting an eukaryotic cell and antiviral mechanism of metal nanoparticles.

In the crusade toward the development of drugs for the therapy of viral diseases, the emergence of resistant viral strains and adverse side effects associated with a prolonged use represent huge obstacles that are difficult to circumvent. Therefore, multidisciplinary research efforts, integrated with classical epidemiology and clinical approaches, are crucial for the development of improved antivirals through alternative strategies. Nanotechnology has emerged giving the opportunity to re-explore biological properties of known antimicrobial compounds, such as metals, by the manipulation of their sizes. Metal nanoparticles, especially the ones produced with silver or gold, have proven to exhibit virucidal activity against a broad-spectrum of viruses, and surely to reduce viral infectivity of cultured cells. In most cases, a direct interaction between the nanoparticle and the virus surface proteins could be demonstrated or hypothesized. The intriguing problem to be solved is to understand the exact site of interaction and how to modify the nanoparticle surface characteristics for a broader and more effective use. Besides the direct interaction with viral surface glycoproteins, metal nanoparticles may gain access into the cell and exert their antiviral activity through interactions with the viral genome (DNA or RNA). Furthermore, the intracellular compartment of an infected cell is overcrowded by virally encoded and host cellular factors that are needed to allow viral replication and a proper production of progeny virions. The interaction of metal nanoparticles with these factors, which are the key to an efficient viral replication, may also represent a further mechanism of action (Figure 2).

Most of the published literature describes the antiviral activity of silver or gold nanoparticles against enveloped viruses, with both a DNA or an RNA genome. Considering that one of the main arguments toward the efficacy of the analysed nanoparticles is the fact that they in virtue of their shape and size, can interact with virus particles with a well-defined spatial arrangement, the possibility of metal nanoparticles being active against naked viruses seems appealing. Moreover, it has been already proven that both silver and gold nanoparticles may be used as a core material. However, no reports are yet available for the use of other metals, but the future holds many surprises, especially considering that the capping molecules that could be investigated are virtually unlimited.

Nonetheless, for metal nanoparticles to be used in therapeutic or prophylactic treatment regimens, it is critical to understand the *in vivo* toxicity and potential for long-term sequelae associated with the exposure to these compounds. Additional research is needed to determine how to safely design, use, and dispose products containing metal nanomaterials without creating new risk to humans or the environment.

## Figures and Tables

**Table 1:** Antiviral metal nanoparticles.

Virus	Family	Metal Nanoparticle Composition (size)	Mechanism of Action	References
Human immunodeficiency virus type 1 (HIV-1)	Retroviridae	PVP-coated silver nanoparticles (1–10 nm)	Interaction with gp120	[38,39,40]
Herpes simplex virus type 1 (HSV-1)	Herpesviridae	MES-coated silver and gold nanoparticles (4 nm)	Competition for the binding of the virus to the cell	[43,44]
Respiratory syncytial virus	Paramyxoviridae	PVP-coated silver nanoparticles (69 nm +/− 3 nm)	Interference with viral attachment	[42]
Monkeypox virus	Poxviridae	Silver nanoparticles and polysaccharide-coated Silver nanoparticles (10–80 nm)	Block of virus-host cell binding and penetration	[45]
Influenza virus	Orthomyxoviridae	Sialic-acid functionalized gold nanoparticles (14 nm)	Inhibition of virus binding to the plasma membrane	[46]
Tacaribe virus (TCRV)	Arenaviridae	Silver nanoparticles and polysaccharide-coated Silver nanoparticles (10 nm)	Inactivation of virus particles prior to entry	[47]
Hepatitis B virus (HBV)	Hepadnaviridae	Silver nanoparticles; (10–50 nm)	Interaction with double-stranded DNA and/or binding with viral particles	[41]
